# Supplementary material for: EnzML: multi-label prediction of enzyme classes using InterPro signatures
Source: BMC Bioinformatics. 2012 Apr 25;13:61. doi: 10.1186/1471-2105-13-61 (PMC3483700; doi:10.1186/1471-2105-13-61)
Supplement: Addtional file 5 — The Java code to format the data files, evaluate and predict. The file enzml_java_code.tar.gz contains the Java code used to format database data to ARFF and XML formats, to execute cross and train-test (jackknife) evaluations and to record evaluation results to database. More information is included in the readme.txt file and the Javadoc files. The code can be used with a MySQL database. To use a different database software, other JDBC drivers might be required. [file 1471-2105-13-61-S5.gz › java_code/utils/doc/uk/ac/ed/inf/utils/database/class-use/DbManaged.html]

Uses of Class uk.ac.ed.inf.utils.database.DbManaged


---


|  |  |  |
| --- | --- | --- |
| **Overview** \| **Package** \| **Class** \| **Use** \| **Tree** \| **Deprecated** \| **Index** \| **Help** | |  |
| PREV   NEXT | **FRAMES**    **NO FRAMES**     **All Classes** |  |


---


## **Uses of Class uk.ac.ed.inf.utils.database.DbManaged**

| Packages that use DbManaged | |
| --- | --- |
| **uk.ac.ed.inf.utils.database** |  |

Uses of DbManaged in uk.ac.ed.inf.utils.database

| Subclasses of DbManaged in uk.ac.ed.inf.utils.database | |
| --- | --- |
| `class` | `DbCreator`             Class |
| `class` | `DbReader<T,U>`             Class to read from a database. |
| `class` | `DbWriter`             To write to a database table |
| `class` | `TableCreator`             Class to create a table object in the database. |
| `class` | `TableManaged`             TableManaged is a class which has a `TableManager` (an object that coordinates its table activities) |
| `class` | `TableManager`             Class to create/read/write to a database table. |
| `class` | `TableReader`             Class |
| `class` | `TableRow`             Contains the data for a generic row (a list of [table column, value] entries) to be written to a database table. |
| `class` | `TableWriter`             A TableWriter object contains all the information to create, fill and delete a database table. |

---


|  |  |  |
| --- | --- | --- |
| **Overview** \| **Package** \| **Class** \| **Use** \| **Tree** \| **Deprecated** \| **Index** \| **Help** | |  |
| PREV   NEXT | **FRAMES**    **NO FRAMES**     **All Classes** |  |


---
